# Aerobic Physical Exercise Improves Exercise Tolerance and Fasting Glycemia Independent of Body Weight Change in Obese Females

**DOI:** 10.3389/fendo.2021.772914

**Published:** 2021-12-14

**Authors:** Daniela Boschetti, Cynthia R. Muller, Anna Laura V. Américo, Bruno Vecchiatto, Luiz Felipe Martucci, Renata O. Pereira, Cláudia P. Oliveira, Patricia Fiorino, Fabiana S. Evangelista, Anna Karenina Azevedo-Martins

**Affiliations:** ^1^ School of Arts, Science and Humanities, University of Sao Paulo, São Paulo, Brazil; ^2^ Department of Bioengineering, University of California San Diego, La Jolla, San Diego, CA, United States; ^3^ Department of Experimental Pathophysiology, Faculty of Medicine, University of São Paulo, São Paulo, Brazil; ^4^ Translational Medicine Division, Department of Medicine, Federal University of São Paulo, Sao Paulo, Brazil; ^5^ Division of Gastroenterology and Hepatology, Department of Gastroenterology (LIM 07), Faculty of Medicine, University of São Paulo, São Paulo, Brazil; ^6^ Renal, Cardiovascular and Metabolic Physiopharmacology Laboratory, Health and Biological Science Center, Mackenzie Presbyterian University, São Paulo, Brazil

**Keywords:** obesity, insulin resistance, physical exercise, female, leptin deficiency

## Abstract

Obesity is associated with increased risk of several chronic diseases and the loss of disease-free years, which has increased the focus of much research for the discovery of therapy to combat it. Under healthy conditions, women tend to store more fat in subcutaneous deposits. However, this sexual dimorphism tends to be lost in the presence of comorbidities, such as type 2 diabetes mellitus (T2DM). Aerobic physical exercise (APE) has been applied in the management of obesity, however, is still necessary to better understand the effects of APE in obese female. Thus, we investigated the effect of APE on body weight, adiposity, exercise tolerance and glucose metabolism in female ob/ob mice. Eight-weeks-old female wild-type C57BL/6J and leptin-deficient ob/ob mice (Lep^ob^) were distributed into three groups: wild-type sedentary group (Wt; n = 6), leptin-deficient sedentary group (Lep^ob^S; n = 5) and leptin-deficient trained group (Lep^ob^T; n = 8). The Lep^ob^T mice were subjected to 8 weeks of aerobic physical exercise (APE) at 60% of the maximum velocity achieved in the running capacity test. The APE had no effect in attenuating body weight gain, and did not reduce subcutaneous and retroperitoneal white adipose tissue (SC-WAT and RP-WAT, respectively) and interscapular brown adipose tissue (iBAT) weights. The APE neither improved glucose intolerance nor insulin resistance in the Lep^ob^T group. Also, the APE did not reduce the diameter or the area of RP-WAT adipocytes, but the APE reduced the diameter and the area of SC-WAT adipocytes, which was associated with lower fasting glycemia and islet/pancreas area ratio in the Lep^ob^T group. In addition, the APE increased exercise tolerance and this response was also associated with lower fasting glycemia in the Lep^ob^T group. In conclusion, starting APE at a later age with a more severe degree of obesity did not attenuate the excessive body weight gain, however the APE promoted benefits that can improve the female health, and for this reason it should be recommended as a non-pharmacological therapy for obesity.

## Introduction

Obesity is characterized by an exaggerated accumulation of body fat in white adipose tissue (WAT) accompanied by a different distribution in its body deposits, implying the increase in the visceral WAT/subcutaneous WAT ratio. Under healthy conditions, women tend to store more fat in subcutaneous deposits, while men are prone to greater visceral fat deposition (1). However, in the presence of comorbidities, such as type 2 diabetes mellitus (T2DM), this sexual dimorphism tends to be lost, so that 70% of women with T2DM have visceral obesity versus 40% of men (2). It is no coincidence that in both women and men, the new distribution of WAT in obesity is associated with increased risk of several chronic diseases and the loss of disease-free years (3).

The percentage of visceral WAT in obese subjects has a strong correlation with insulin resistance and deficient control of glucose metabolism, which in turn increases the risk of cardiovascular disease (4–6). The higher percentage of visceral WAT is followed by an alteration in the profile of adipokines produced by the individual. Also, the WAT of an obese person shows infiltration with lymphocytes and macrophages and characteristics of a subclinical inflamed tissue (7). Although the mechanism involved in increasing cardiovascular risk are the same in both sexes, it has been demonstrated that women showed a risk of death from cardiovascular disease 1.5 times higher than men. When diagnosed with obesity and T2DM, the risk of myocardial infarction followed by death was 3 to 6 times higher (8).

Different strategies have been applied in the management of obesity and the prescription of aerobic physical exercise (APE) has been very efficient for this purpose. In this sense, there is already much evidence to support the notion that in overweight or obese individuals, the later physical activity is incorporated into the routine, the higher the metabolic cost, as numerous health-related variables worsen in short periods, especially in those who already have metabolic damage (for review, please read reference 9). Among the many benefits of APE, are the reduction in adiposity and improvement in glucose metabolism (9–12), reduction in pro-inflammatory adipokines and higher secretion of anti-inflammatory ones (adiponectin and IL-10) (13), and the increase in exercise tolerance regardless of weight loss (14). Regarding the sex, both males and females respond positively to APE for the control of blood glucose (15, 16), body mass (17, 18), blood pressure (19) and lipid profile (20, 21), which are crucial to maintaining cardiovascular health. But the magnitude of response, the type of exercise and the time taken to obtain the benefits should differ between males and females, mainly due to the female hormones (22).

The studies performed with women or female models is increasing, however it is still necessary to better understand the effects of APE in obese female (23). Considering APE benefits to the characteristics of the female organism, the purpose of this study was to investigate the effect of APE on body weight, adiposity, exercise tolerance and glucose metabolism in female ob/ob mice, a rodent lineage lacking in leptin secretion that presents a phenotype of obesity at birth.

## Materials and Methods

### Animals

Eight-week-old female wild-type C57BL/6J and leptin-deficient mice (Lep^ob^) were obtained from the Laboratório de Gastroenterologia Clínica e Experimental (LIM-07) of University of São Paulo. The animals were separated into three groups: wild-type sedentary group (Wt; n = 6), leptin-deficient sedentary group (Lep^ob^S; n = 5) and leptin-deficient trained group (Lep^ob^T; n = 8). Animals were kept under the same housing conditions (12- h light/12- h dark cycle, temperature 22 ± 2°C) with water and food *ad libitum.* All procedures were approved by the Ethics Committee of School of Arts, Sciences and Humanities of University of São Paulo (# 001/2017). The *in vivo* evaluations were conducted during non-ovulatory phase of the estrous cycle.

### Aerobic Physical Exercise

Animals were submitted to aerobic physical exercise (APE) during the dark cycle (i.e., during their active period) on a motorized treadmill for 1 h/day at 60% of maximal speed achieved in the running capacity test (speed range between 0.6 km/h and 0.8 km/h), five times per week for eight weeks (24). The duration was progressively increased, starting with 30 minutes in the first week and reaching 60 minutes in the fourth week. This training duration was maintained until the end of the protocol. To minimize the influence of the treadmill stress, Wt and Lep^ob^S mice were placed on the treadmill for 5 min twice weekly at 0.2 km/h.

### Exercise Tolerance Test

Running capacity was assessed before, in the fourth and in the eighth week of APE using a progressive test without incline on a treadmill until exhaustion as described by Ferreira et al. (24). The initial speed was 0.4 km/h and the speed was increased by 0.2 km/h every three minutes until exhaustion of the animal, which was characterized by the impossibility of maintaining the standard rate. In the 4th week of the protocol, the test was assessed only in groups trained to readjust the intensity of APE.

### Indirect Calorimetry

In the eighth week, the animals were acclimatized in the Oxylet Calorimetry System (Panlab, Barcelona, Spain) and the measurements were done during rest. Firstly, the animals were fasted (2-h) and then the volumes of oxygen consumption (VO_2_) and carbon dioxide production (VCO_2_) were measured during 30 min of resting. The non-protein respiratory exchange ratio (RER), a measurement of metabolic substrate preference, was calculated as the molar ratio of VCO_2_ to VO_2_. Energy expenditure (EE) was calculated by the formula [3.815 + (1.232 x R)] x VO_2_ x 1.44 and the result was expressed as kcal.Kg^-1^.min^-1^. The rate of oxidation of carbohydrate (CHO) and lipids (LIP) were calculated using the formulas (25): CHO = (4.55 x VO_2_) - (3.21 x VCO_2_) and LIP= (1.67 x VO_2_) - (1.67 x VCO_2_). Both data were expressed as mg.min-^1^.kg^-1^.

After the resting measurement, the animal was subjected to the exercise tolerance test using the protocol described previously. The volumes of VO_2_ and VCO_2_ were continuously measured until the animal reaches the exhaustion. The maximum VO_2_ (VO_2_max) and VCO_2_ (VCO_2_max) were considered the average obtained in the last stage of the test. The running intensity at which VO_2_max was reached (iVO2max) was measured as described by Machado et al. (26). The results were expressed as mg.min^-1^.kg^-1^.

### Body Weight and Food Intake

The animals were weighed weekly on a digital scale (Gehaka/model BG4001, São Paulo, Brazil), on the same day and time. In addition to the evolution of body weight, we calculated the body weight gain through the difference between final body weight (week 8) and initial body weight (week 0). The 24-h food intake was determined weekly throughout the study in mice’s groups that were housed in the same cage.

### Glucose Tolerance Test (GTT) and Insulin Tolerance Test (ITT)

Fasting glycemia, GTT and ITT were performed after the 8-week of APE protocol. The experiments were done in awake animals after an 8-h fast. The glucose load (1 g/kg body weight) was injected as a bolus intraperitoneally, and the blood glucose levels were determined in caudal blood sampled at 0, 15, 30, 60, 90 and 120 min after glucose infusion. The glucose concentration was determined using a glucometer (AccuChek Advantage Roche Diagnostics). After 72 h of GTT test, a similar procedure was performed for ITT. The insulin load (0.75 U/kg body weight) was injected as a bolus intraperitoneally, and the blood glucose levels were determined in caudal blood samples collected at 0, 5, 10, 15, 20, 25 and 30 min after injection. The values obtained between 5 and 30 min were used to calculate the rate constant for the disappearance of plasma glucose (kITT) according to the method proposed by Bonora et al. (27).

### Tissue and Blood Collection

Forty-eight hours after the end of the last APE session, the animals were sacrificed with an intraperitoneal injection of pentobarbital sodium (4 mg/100 g body weight) following exanguination. The animal was weighed and then the subcutaneous (inguinal) and visceral (retroperitoneal) WAT fat pads (SC-WAT and RP-WAT, respectively), interscapular brown adipose tissue (iBAT), skeletal muscles (gastrocnemius, soleus and plantaris) and pancreas were harvested and weighed. The splenic portion of the pancreas was used for the histological analysis.

### Histological Analysis

The morphology of adipocytes was measured in paraffin sections of SC-WAT and RP-WAT fat pads (5μm) stained with hematoxylin and eosin (Sigma). Digital images from 50 adipocytes per animal were obtained using a light microscope (Axio observator. A1, Zeiss, Jena, Germany), at 400x magnification. After digitalization, adipocyte diameter and area were traced and calculated using a computerized morphometric analysis system (Image Pro-Plus 4.1; Media Cybernetics, Silver Spring, MD, USA). In addition, the splenic portion of pancreas included in paraffin was cut (4 μm) and stained with hematoxylin and eosin (Sigma). The images of pancreatic islets and pancreas were acquired in a digital light microscopy (Axio observator. A1, Zeiss, Jena, Germany), at 400x and 100x magnification, respectively. The pancreatic islets area (AI) and the islet/pancreas area ratio (AI/AP) were analyzed in the Image Pro-Plus 4.1 program (Media Cibernetics Inc, Rockville, USA). All analysis was done by a single observer (Boschetti D) blinded to mice identities.

### Statistical Analyses

All values are expressed as mean ± SEM. Data were analyzed with one-way or two-way analyses of variance (ANOVA). The Tuckey *post hoc* test was used to determine differences between means when a significant change was observed using ANOVA. Pearson correlation was used to analyze the association between variables. *p* value equal to or less than 0.05 was statistically significant (GraphPad Prism, v.7.0).

## Results

### Body Weight, Tissues Weight and Food Intake

**Figure 1:**
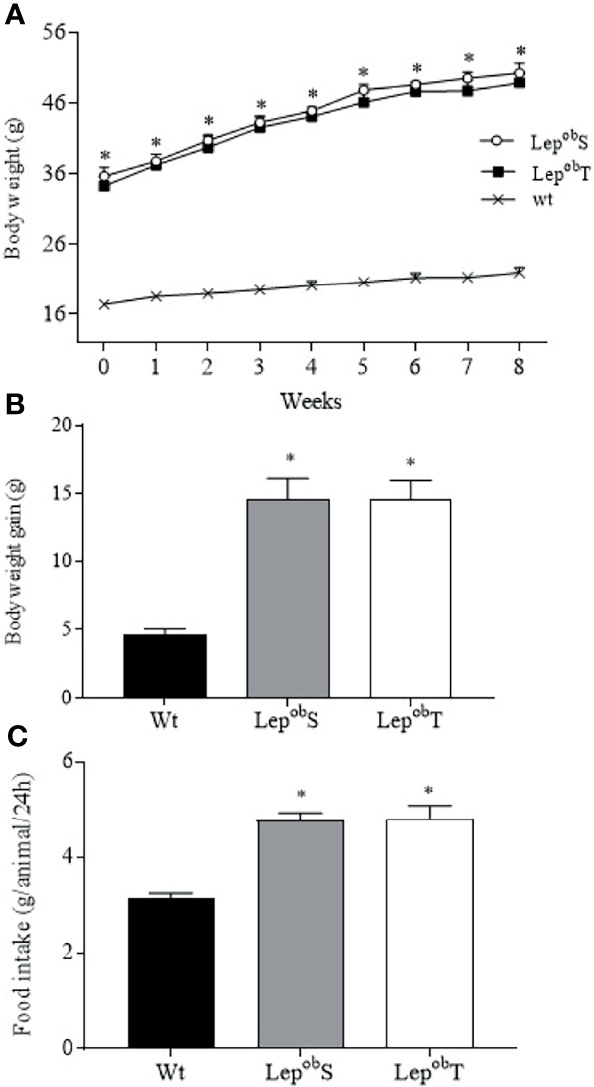
Body weight evolution **(A)**, body weight gain **(B)**, and food intake **(C)**. Wt (n = 6), Lep^ob^S (n = 5) and Lep^ob^T (n = 8). Error bars indicate the SEM. *p ≤ 0.05 vs. Wt. The results of body weight evolution were compared by two-way ANOVA and body weight gain and food intake by one-way ANOVA plus Tuckey *post hoc* test.

From the beginning until the end of the experimental protocol, body weight of Lep^ob^S and Lep^ob^T were higher than Wt group (**Figure 1A**). Also, Lep^ob^S and Lep^ob^T groups showed higher body weight gain compared with Wt group (**Figure 1B**). Both results confirmed that APE did not reduce the body weight gain typically observed in Lep^ob^ mice. In addition, both Lep^ob^S and Lep^ob^T groups increased daily food intake compared with Wt group, which revealed that PA did not counteract the hyperphagic response in the Lep^ob^T group (**Figure 1C**).

As shown in **Table 1**, the groups Lep^ob^S and Lep^ob^T increased the weight of both SC-WAT and RP-WAT compared with Wt group. Although the Lep^ob^T group had shown 18% less RP-WAT weight compared to the Lep^ob^S group, this difference was not statistically significant. In addition, the weight of iBAT was higher in the Lep^ob^T group compared with Wt group. Regarding the weight of skeletal muscles, it was observed that both gastrocnemius and plantar muscles of the animals of Lep^ob^S and Lep^ob^T were lighter compared with Wt group, while no difference was found in the soleus muscle. However, the gastrocnemius and soleus muscles were heavier in the Lep^ob^T group when compared with the Lep^ob^S. Finally, there was no statistical difference in the pancreas weight among the groups (**Table 1**).

**Table 1:** The weight of adipose tissue, skeletal muscles, and pancreas.

	Wt (n = 6)	Lep^ob^S (n = 5)	Lep^ob^T (n = 8)
SC-WAT (mg/g)	17.47 ± 2.43	104.07 ± 2.08*	110.11 ± 6.35*
RP-WAT (mg/g)	5.75 ± 1.08	43.52 ± 8.44*	35.63 ± 2.41*
iBAT (mg/g)	3.57 ± 0.49	17.06 ± 4.90	21.00 ± 5.63*
Gastrocnemius (mg/g)	7.93 ± 0.55	2.37 ± 0.18*	3.72 ± 0.2*^#^
Plantar (mg/g)	1.12 ± 0.11	0.33 ± 0.05*	0.59 ± 0.13*
Soleo (mg/g)	0.78 ± 0.14	0.31 ± 0.04	1.08 ± 0.27^#^
Pancreas (mg/g)	20.10 ± 3.89	21.64 ± 8.86	18.85 ± 6.10

Data are presented as mean ± SEM. Wt (n = 6), Lep^ob^S (n = 5) and Lep^ob^T (n = 8). SC-WAT, subcutaneous white adipose tissue; RP-WAT, retroperitoneal white adipose tissue; iBAT, brown adipose tissue. *p ≤ 0.05 vs. Wt; ^#^p ≤ 0.05 vs. Lep^ob^S. The results were compared by the one-way ANOVA plus Tuckey post hoc test.

**Figure 2:**
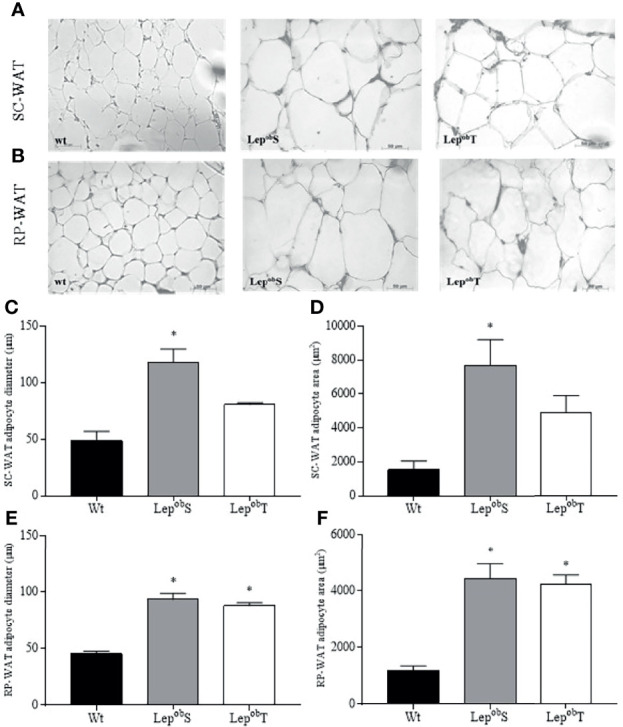
Representative histological sections of SC-WAT **(A)** and RP-WAT **(B)** to visualize the morphology of adipocytes at 400X magnification, SC-WAT adipocyte diameter **(C)**, SC-WAT adipocyte area **(D)**, RP-WAT adipocyte diameter **(E)**, RP-WAT adipocyte area **(F)**. Wt (n = 5), Lep^ob^S (n = 4) and Lep^ob^T (n = 4). Error bars indicate the SEM. *p ≤ 0.05 vs. Wt. The results were compared by the one-way ANOVA plus Tuckey *post hoc* test.

Morphometric analyses of the WAT are shown in **Figure 2**. Increased adipocyte diameter and area were observed in the SC-WAT fat pad in the Lep^ob^S group compared with Wt group (**Figures 2A, C, E**). The APE was efficient in decreasing both the diameter and the area of ​​adipocytes in Lep^ob^T animals compared to Lep^ob^S (**Figures 2A, C, E**). The hypertrophy of adipocyte was also found in the RP-WAT as showed by the higher diameter and area of adipocyte in the Lep^ob^S group compared with Wt group. However, APE did not prevent these responses in the Lep^ob^T animals (**Figures 2A, D, E**).

**Figure 3:**
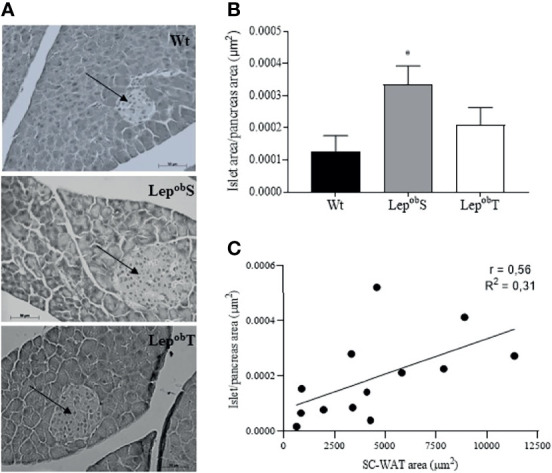
Representative histological sections of pancreas to visualize the morphology of pancreatic islet at 400X magnification **(A)**, pancreatic islet area to pancreas area ration **(B)**, and correlation between pancreatic islet area to pancreas area ratio and SC-WAT adipocytes area **(C)**. Wt (n = 6), Lep^ob^S (n = 5) and Lep^ob^T (n = 5). The arrows point to the pancreatic islets. Error bars indicate the SEM. *p ≤ 0.05 vs. Wt. The results were compared by the one-way ANOVA plus Tuckey *post hoc* test.

We also evaluated pancreatic islet morphometry and although the pancreas weight did not differ statistically among groups, the pancreatic islets of Lep^ob^S (3.2 x 10^5^ ± 1 x 10^5^ µm^2^) were almost three times larger than those in groups Wt (1.24 x1 0^5^ ± 0.98 x 10^5^ µm^2^) and Lep^ob^T (1.21 x 10^5^ ± 0.43 x 10^5^ µm^2^; **Figure 3A**). In addition, the animals in the Lep^ob^S group had a higher islet/pancreas area ratio compared with Wt group, which was countered by APE in the Lep^ob^T group (**Figure 3B**). Also, we observed a positive correlation between islet/pancreas area ratio and SC-WAT adipocytes area (r = 0.56, p = 0.0041) (**Figure 3C**).

### Metabolic Parameters

**Table 2** shows it was showed the results of indirect calorimetry during the rest and at maximal running test. There was no difference in the values of VO_2_, VCO_2_, RER and EE among groups during rest. Also, carbohydrate and lipid oxidation rates during rest were not different among groups. When the metabolic parameters were evaluated during the exercise tolerance test, reduction in VO_2_ max, VCO_2_ max, iVO_2_ and EE in both Lep^ob^S and Lep^ob^T groups was found when compared with Wt group. However, the time until exhaustion (exercise performance) and the running intensity at which VO_2_ max was reached (iVO2) were higher in the Lep^ob^T compared with Lep^ob^S group. These results revealing that the leptin deficiency-induced obesity caused significant damage in the aerobic capacity, but that the APE mitigated this loss in the Lep^ob^T group (**Table 2**).

**Table 2:** Metabolic response at rest and at maximal running test.

	Wt (n = 6)	Lep^ob^S (n = 5)	Lep^ob^T (n = 8)
*Resting*			
VO_2_ (ml.min^-1^.kg^-1^)	36.08 ± 4.77	28.38 ± 3.15	31.07 ± 1.81
VCO_2_ (ml.min^-1^.kg^-1^)	28.96 ± 3.75	22.32 ± 2.37	23.93 ± 1.47
RER	0.81 ± 0.05	0.79 ± 0.04	0.71 ± 0.09
EE (kcal. kg^-1^.min^-1^)	249.58 ± 32.36	195.21 ± 21.22	216.40 ± 13.43
CHO oxidation (mg.min^-1^.kg^-1^)	71.20 ± 11.83	57.45 ± 7.76	64.58 ± 5.06
Lipid oxidation (mg.min^-1^.kg^-1^)	11.89 ± 3.43	10.11 ± 2.13	11.93 ± 1.69
*Running test*			
Exercise performance (minutes)	35.17 ± 1.08	15.20 ± 1.36*	21.25 ± 1.54*^#^
VO_2_ max (ml.min^-1^.kg^-1^)	46.78 ± 2.11	30.10 ± 1.39*	32.22 ± 2.36*
VCO_2_ max (ml.min^-1^.kg^-1^)	45.47 ± 1.81	25.28 ± 0.30*	30.67 ± 2.24*
RER	0.91 ± 0.07	0.91 ± 0.05	0.92 ± 0.05
iVO_2_ (km/h)	2.06 ± 0.06	0.70 ± 0.06*	1.13 ± 0.12*^#^
EE (kcal. kg^-1^.min^-1^)	324.93 ± 20.54	213.67 ± 10.05*	240.70 ± 15.10*
CHO oxidation (mg.min^-1^.kg^-1^)	66.91 ± 11.75	49.53 ± 5.77	49.55 ± 12.59
Lipid oxidation (mg.min^-1^.kg^-1^)	2.19 ± 4.88	4.79 ± 2.70	3.09 ± 5.21

Data are presented as mean ± SEM. VO_2_ , oxygen consumption; VCO_2_ , carbon dioxide production; RER , respiratory exchange ratio; EE , energy expenditure; CHO , carbohydrate; iVO_2_ , running intensity at which VO_2_ max was reached. The results were compared by the one-way ANOVA plus Tuckey post hoc test. *p ≤ 0.05 vs. Wt; ^#^p ≤ 0.05 vs. Lep^ob^S.

**Figure 4:**
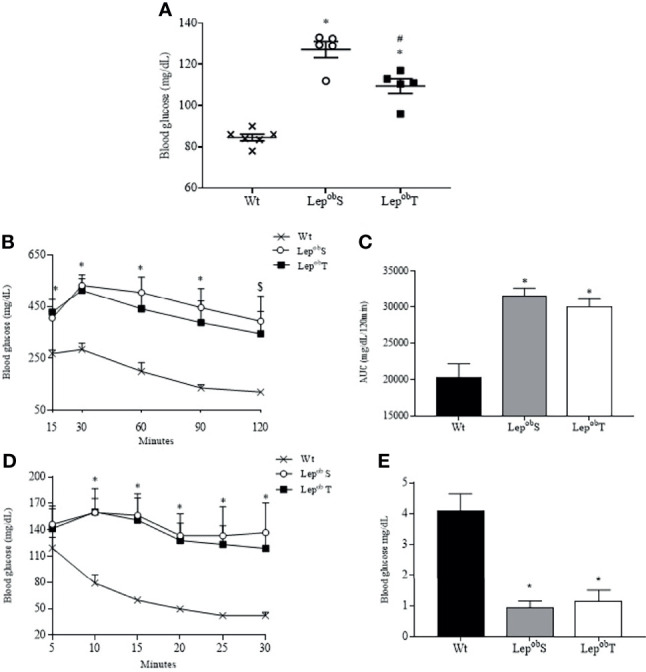
Fasting blood glucose **(A)**, glucose tolerance test **(B)**, area under the curve **(C)**, insulin tolerance test **(D)** and kITT **(E)**. Wt (n = 6), Lep^ob^S (n = 5) and Lep^ob^T (n = 5). AUC = area under the curve, kITT = glucose disappearance constant rate. Error bars indicate the SEM. *p ≤ 0.05 vs. Wt, ^#^p ≤ 0.05 vs. Lep^ob^S, $p ≤ 0.05 Wt vs. Lep^ob^S. The results were compared by the one-way ANOVA (Figure A, C and E) and by the two-way ANOVA (Figure B and D) plus Tuckey *post hoc* test.

Glucose metabolism was evaluated at the end of the experimental protocol, and as shown in the **Figure 4A**, the Lep^ob^S and Lep^ob^T groups increased fasting glycemia (127.2 ± 3.88 mg/dL and 109.5 ± 63.56 mg/dL, respectively) compared with Wt group (84.5 ± 1.61 mg/dL). In addition, fasting glycemia was significantly lower in Lep^ob^T group compared with Lep^ob^S group. Reductions in the glucose tolerance were observed in the Lep^ob^S and Lep^ob^T groups compared with Wt group (**Figures 4B, C**). In the same way, damages in the insulin resistance were found in Lep^ob^S and Lep^ob^T groups compared with Wt group (**Figures 4D, E**).

**Figure 5:**
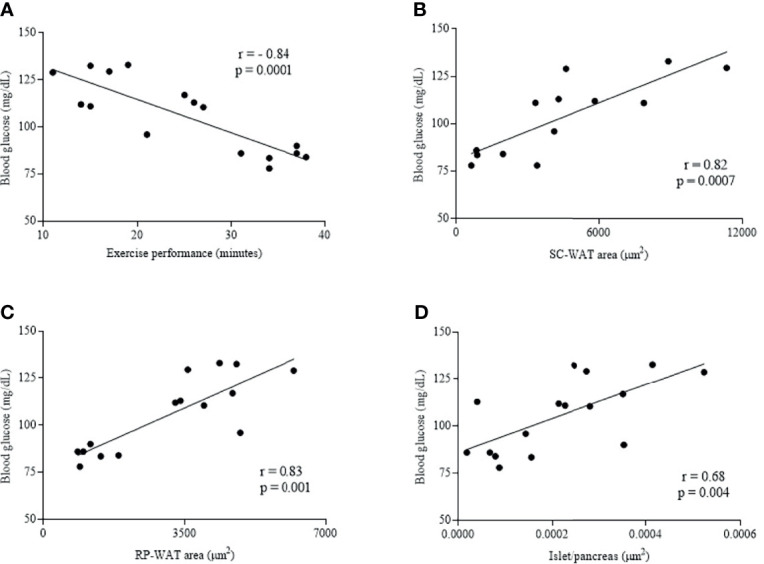
Correlation of fasting blood glucose and exercise performance (minutes) **(A)**, fasting blood glucose and SC-WAT adipocytes area **(B)**, fasting blood glucose and RP-WAT adipocytes area **(C)** and fasting blood glucose and islet area to pancreas area ratio **(D)**. Wt (n = 5), Lep^ob^S (n = 4-5) and Lep^ob^T (n = 4-5).

A negative correlation was observed between fasting blood glucose and exercise performance (minutes) (r = -0.84, p = 0.0001) (**Figure 5A**). A positive correlation was observed between fasting blood glucose and SC-WAT adipocytes area (r = 0.82, p = 0.0007) (**Figure 5B**), fasting blood glucose and RP-WAT adipocytes area (r = 0.83, p = 0.001) and fasting blood glucose and islet area to pancreas area ratio (r = 0.68, p = 0.004) (**Figures 5C, D**).

## Discussion

The growing number of obese women and the negative repercussions for health has motivated the search for understanding the prevention and treatment of obesity. Despite that our group previously reported a reduction in body weight gain in male ob/ob mice with the same age and exercise protocol as applied here (28, 29), we revealed that APE did not avoid the excessive body weight gain in female ob/ob mice. We also observed that females ob/ob of the present study seem to gain more weight than males from our previous study (28, 29). The sex-dependent obesity response in ob/ob mice has been characterized in studies with metabolomic and lipidomic analysis (30, 31), and our research adds novel knowledge by suggesting that the APE-mediated benefits on the body weight of ob/ob mice is also sex dependent, which reinforces the importance of considering the differences between the sexes for the management of body weight. However, further studies are still needed to unravel such mechanisms.

The reduction of body weight has been shown in female ob/ob mice when the APE started earlier at five or six weeks of age (32, 33). In our study the animals started the exercise protocol at eight weeks old (with severe degree of obesity), which could, in part, explain the discrepancy between our and other results. The adiposity negatively influences the skeletal muscle function, including damage to force generation, gait speed and locomotor pattern (34, 35), which can increase the difficulty of exercising and early exhaustion. In this sense, our APE protocol was enough to promote some beneficial adaptations, however the earlier start of the APE may be more efficient for the control of body weight.

The unchanged body weight gain in the Lep^ob^T group also can be explained by the maintenance of hyperphagia. The body weight stems from caloric intake and energy expenditure. In turn, energy expenditure is determined by resting and exercise energy expenditures and thermogenic effect of food. As observed in the results, the resting energy expenditure was not different among the groups, and despite the resting measurement consider only a short period of 24h and we did not evaluate of thermogenic effect of food, it is possible that the energy expenditure promoted by APE was not enough to reduce body weight gain because it was compensated by the high caloric intake of Lep^ob^T group. According to the literature, the effect of APE on food consumption is controversial since the authors showed increases (36) or no changes in food consumption (12, 37). In this sense, our results allow us to suggest that for severe obesity cases, weight control will be efficient if the practice of physical exercise is associated with caloric restriction.

The reduction of WAT can improve insulin sensitivity and reduce the risk of T2DM (9, 12). Here, the APE decreased the diameter and the area of SC-WAT adipocytes in Lep^ob^T compared to Lep^ob^S, which may have contributed to the better fasting glycemia after APE. In fact, we observed a positive correlation between SC-WAT adipocytes area and fasting blood glucose. Stanford et al. (10) showed that transplanting SC-WAT from exercise-trained mice in sedentary ones improved glucose tolerance and insulin sensitivity. They also observed that the deleterious effects of high-fat on glucose homeostasis were completely reversed in high-fat fed mice transplanted with SC-WAT from exercise-trained mice. On the other hand, we did not find changes in the diameter and the area of RP-WAT adipocytes, but a positive correlation between RP-WAT adipocytes area and fasting blood glucose. These data reinforce the idea that APE induced depot-specific effects, which was previously observed in genes involved in mitochondrial activity, glucose metabolism, and fatty acid uptake and oxidation by Lehnig et al. (2019) (11).

Leptin deficiency is associated with reduction in the sympathetic innervation of BAT, which results in damage of oxidative capacity and thermogenesis (38, 39). In a thermogenesis-reduced state, the conversion of brown adipocyte to white-like unilocular cells (BAT whitening) associated with lower lipid oxidation results in increased BAT (40). Although the APE is a stimulus that counteracts this typical BAT phenotype in obesity, our results showed that the APE was not efficient to reverse the high weight of the iBAT in the Lep^ob^T animals.

While metabolic diseases are associated with damages in energy metabolism and reduced aerobic capacity, the greater the aerobic capacity, the lower is the risk of cardiovascular and metabolic diseases (41). The APE reduced the negative effect of leptin deficiency in the Lep^ob^T group by increasing the exercise performance, the running intensity at which VO_2_max was reached (iVO_2_max), and the weight of soleus and gastrocnemius muscles. Furthermore, we found a negative correlation between fasting blood glucose and exercise performance (minutes), which reinforces that APE is efficient to improve glucose metabolism and that is a good therapeutic strategy to ameliorate metabolic diseases. We also expected that Lep^ob^T group could improve VO_2_max, however additional studies are necessary to understand if the VO_2_max result is a specific pattern of female ob/ob mice and/or is due to the exercise protocol used in the treadmill test.

Since leptin contributes to the maintenance of glucose homeostasis, ob/ob mice showed damages in glycemic control. In addition, the islet size in ob/ob mice can be up to ten times higher than in control mice, depending on the age (42). Here, we found greater islet/pancreas area ratio, hyperglycemia, glucose intolerance and insulin resistance in the Lep^ob^S compared with Wt group. The APE decreased the islet/pancreas area ratio and hyperglycemia in the Lep^ob^T group without changes in glycemic tests. These results are different from the study of Jimenez-Maldonado et al. (43), in which it was observed that healthy male rats trained with high or moderate intensity APE showed β-cells hypertrophy, while the moderate intensity increased the number of β-cells per islet, without change on islet/pancreas area ratio.

Interesting that the positive correlation between islet/pancreas area ratio and fasting blood glucose and islet/pancreas area ratio and SC-WAT adipocytes area revealed that the smallest islet size was associated with the lower blood glucose levels and SC-WAT adipocytes size. Furthermore, considering that increasing in islet size is much more due to the elevated demand for insulin than the leptin deficiency (44) and the reduction of fasting glycemia, it is possible that APE has improved the glycemic control in an insulin-independent manner by the activation of AMP-activated protein kinase (AMPK) in the skeletal muscle (45), and therefore reduced the islets workload of the ob/ob animals. Further study is still needed to investigate the effect of AET on AMPK in ob/ob mice.

In conclusion, the results provide evidence that starting APE at a later age with a more severe degree of obesity did not avoid the excessive body weight gain in female ob/ob mice. However, the APE had positive effects in reducing SC-WAT adipocytes size which was associated with lower fasting glycemia and islet/pancreas area ratio. In addition, the APE increased exercise tolerance and this response was also associated with lower fasting glycemia. Thus, despite not changing the weight value on the scale, the training promoted benefits that can improve the female health, and for this reason it should be recommended as a non-pharmacological therapy for obesity.

## Data Availability Statement

The original contributions presented in the study are included in the article/supplementary material. Further inquiries can be directed to the corresponding author.

## Ethics Statement

The animal study was reviewed and approved by Ethics Committee of School of Arts, Sciences and Humanities of University of São Paulo (protocol number 001/2017).

## Author Contributions

Conceptualization and design: AA-M, FE, and CO. Data collection and analysis: CM, AA, BV, LM, RP, and PF. Data curation: AA-M and FE. Manuscript drafting: DB, AA-M, and FE. Supervision: AA-M and FE. All authors contributed to the article and approved the submitted version.

## Funding

DB held a scholarship from Coordenação de Aperfeiçoamento de Pessoal de Nível Superior (CAPES). This study was supported by São Paulo Research Foundation (FAPESP) to FE (2015/04948-4).

## Conflict of Interest

The authors declare that the research was conducted in the absence of any commercial or financial relationships that could be construed as a potential conflict of interest.

## Publisher’s Note

All claims expressed in this article are solely those of the authors and do not necessarily represent those of their affiliated organizations, or those of the publisher, the editors and the reviewers. Any product that may be evaluated in this article, or claim that may be made by its manufacturer, is not guaranteed or endorsed by the publisher.
